# Effects of Grazing on the Behaviour, Oxidative and Immune Status, and Production of Organic Dairy Cows

**DOI:** 10.3390/ani9060371

**Published:** 2019-06-18

**Authors:** Antonino Di Grigoli, Adriana Di Trana, Marco Alabiso, Giuseppe Maniaci, Daniela Giorgio, Adriana Bonanno

**Affiliations:** 1 Department of Agricultural, Food and Forest Sciences (SAAF), University of Palermo, Viale delle Scienze n. 13, 90128 Palermo, Italy; marco.alabiso@unipa.it (M.A.); giuseppe.maniaci@unipa.it (G.M.); adriana.bonanno@unipa.it (A.B.); 2 School of Agricultural, Forestry, Food and Environmental Sciences (SAFE), Viale dell’Ateneo Lucano n. 10, 85100 Potenza, Italy; adriana.ditrana@unibas.it (A.D.T.); giorgio_daniela@alice.it (D.G.)

**Keywords:** organic dairy farm, behaviour, oxidative status, immune response, milk fatty acid composition

## Abstract

**Simple Summary:**

In European organic dairy farms, the use of grazing appears to be a controversial topic. The laws that regulate the sector do not indicate in an incontrovertible way the obligation to provide grazing; accordingly, organic farmers sometimes allow dairy cows access to only an open air fenced area rather than to a grazing pasture. This work confirms the validity and benefits of grazing, compared to access to an outdoor space, in terms of the behaviour and milk production of dairy cows.

**Abstract:**

This study compared the effects of a short daily grazing time with those of permanent free-stall housing on the behaviour, oxidative status, immune response, and milk production of organically reared cows. During a 63-day period, two homogeneous groups of eight lactating Brown cows were allocated to either housing (H) in a free-stall building for 24 h/day. Feeding was based on a total mixed ration or grazing (G) on barley grass for 5 h/day, and housing in a free-stall structure with feeding was based on the same total mixed ration offered to the H group. With regard to behaviour, H cows spent more time idling, walking, drinking, and self-grooming, whereas G cows showed a greater intent to eat and interact socially. Moreover, G cows exhibited slightly higher reactive oxygen metabolites and similar biological antioxidant potential concentrations than the H group, which indicates that short grazing resulted in an almost negligible increase in oxidative stress and an unchanged antioxidant capacity. Skin tests, performed by injecting phytohemoagglutinin intradermally, indicated that G cows had thicker skin than H cows at the end of the trial, an index of a better cell-mediated immune response. Grazing did not affect milk yield but improved milk quality in terms of an increase in fat and a reduction in urea content, somatic cell count, and total microbial count. Milk from G cows was richer in saturated fatty acids, likely because of the contribution of palmitic acid present in the grazed barley grass, and also showed higher contents of some healthy fatty acids, such as rumenic acid and α-linolenic acid, and a lower omega-6/omega-3 ratio. These results show that including a short grazing time in the diets of organic dairy cows does not have negative consequences for milk production and contributes to improved milk quality as well as to a more efficient immune response in the cows.

## 1. Introduction

In the past few years there has been a significant increase in the number of organic farms in Italy. Thus, it is becoming increasingly important to update the technical support for organic livestock farming, especially in relation to animal feeding. For example, one problem observed in herds of high-producing dairy cows is the reduction of milk yield which is attributed mainly to unmet feeding requirements prescribed for organic livestock [1].

The question of feeding in organic livestock farming also includes grazing. European Community Regulation 2007/834 promotes direct access to pasture among animals (Article 14: “… livestock shall have permanent access to open air areas, preferably pasture … with the exception of bees, livestock shall have permanent access to pasture or roughage”). Therefore, in organic livestock farming grazing is recommended but not obligatory, although some countries in northern Europe (e.g., Norway, Sweden and Finland) have passed laws that require dairy cows to have access to pasture in summer, for a period ranging from six weeks to four months [2].

Most people living in developed countries consider the permanent housing of cows to be unacceptable and require animal products to be from farms where animals have good welfare and they can engage in species-specific behaviour. Modern consumers are willing to pay more for products obtained from livestock raised under welfare-positive conditions [3]. In this context, grazing is an important factor for dairy cows to express their natural behaviour and maintain their health [4]. The positive effects of grazing on animals are often related to better functioning of the immune system [5,6] as well as to improved oxidative status. The latter is due to the high number of antioxidants in green forage and the greater physical exercise performed by grazing animals [7]. Moreover, the direct use of forage through grazing is more convenient in economic terms, as it reduces the costs of feeding and human recourses. Grazing also affects the quality of dairy products, resulting in more unsaturated fatty acids (FA), including conjugated linoleic acids (CLA) and omega-3 FA [8], and vitamins [9], making them healthier for the final consumer.

Despite these advantages, modern farms with high-producing dairy cows often do not practice grazing to optimise forage use and control the dietary intake of the animals [10,11]. In particular, such farms avoid grazing because of (i) the uncertainty of pasture production during the year due to changes in thermo-pluviometric conditions; (ii) the high nutritional requirements of modern high-producing dairy cows, which are often not satisfied by grazing; and (iii) the increasingly frequent use of automatic milking systems.

Therefore, this study investigated the validity and benefits of grazing by comparing the behaviour, oxidative status, immune response, and milk yield and quality (with particular reference to FA content) of organic cows that had access to pasture for part of the day with those of cows that were housed in a free-stall system with access to an outdoor area only.

## 2. Materials and Methods

### 2.1. Animals, Feeding Systems, and Management

This experiment was conducted in an organic dairy farm located 650 m above sea level in the province of Palermo (Sicily, Italy; 37°54′ N, 13°55′ E) for 63 days from mid-March to mid-May.

A total of 16 Italian Brown lactating cows, initially 86 ± 7 days in milk and producing 28.5 ± 2.7 kg/day of milk, were divided into two groups of eight cows homogeneous for milk yield and days in milk and assigned to different feeding systems. Cows in the housing group (H) were kept in a free-stall building for 24 h/day, whereas cows in the grazing group (G) grazed a barley grass pasture of 3 ha adjacent to the barn for 5 h/day, from 9:00 h to 14:00 h, and they were kept in a free-stall building. Both groups were fed the same diet indoors based on a total mixed ration (TMR), composed of hay and concentrate (Table 1) offered at 9:00 h to H cows and at 14:00 h to G cows when they returned from the pasture. At 15:00 h, H cows received supplements of soybean (1.0 kg/day) and faba bean (0.5 kg/day), and hay ad libitum. Feed intake was only recorded indoors.

The free-stall barns where cows in both groups were housed contained an indoor resting area with straw-bedded cubicles and a large outdoor area (10 m^2^ per head), which was more than required by Commission Regulation No. 889/2008 on organic livestock. All cows involved in the research had had previous experience with both free-stall housing and grazing, and during the trial they showed no signs of illness. Cows were managed according to Directive 2010/63/EU on the protection of animals used for scientific purposes and the trial complied with the Italian legislation on animal care (DL n. 26, 4 March 2014).

### 2.2. Measurement, Sampling, and Analytical Methods

All measurements and sampling were performed, after a three-week adaptation period, four times during the experimental period at 14-days intervals.

The forage biomass available in the pasture was estimated by cutting five areas of 2.5 m^2^ at ground level; in order to determine the amount of dry matter (DM), samples were collected and then dried at 105 °C until constant weight. Samples of fresh forage ingested by cows at pasture were collected by hand plucking parts of plants after grazing was observed and recorded. The samples collected at pasture and samples of other feed given in the feeder were analysed for the determination of DM, crude protein (N × 6.25), ether extract and ash using AOAC methods [12], and structural carbohydrates such as neutral detergent fibre (NDF), acid detergent fibre (ADF), and acid detergent lignin (ADL) [13]. The chemical composition of the feed used in the diets is reported in Table 1.

The behaviour of cows in the pasture and the barn was observed. There were two observers per experimental group, one positioned inside and the other outside of the barn (using binoculars when necessary). Observations of behaviour were made every 15 min over a 9-h period (from 8:00 h to 17:00 h) using scan sampling, which consisted of a quick observation of the animal’s behaviour and the recording of data on posture (standing or laying) and activity (grazing, eating indoors, drinking, ruminating, walking, idling (eyes opened or closed, but no other activity)). In addition, social interactions (sniffing or rubbing another subject unaggressively), aggressive interactions (butting, pushing, or threatening), and self-grooming were recorded continuously during the 9-h observation period.

Individual blood samples from fasted cows were collected in the morning by coccygeal venipuncture. Plasma oxidants and antioxidant capacity were measured with two different commercial kits (Diacron, Grosseto, Italy) by determining reactive oxygen metabolites (ROMs), mainly hydroperoxides generated by the oxidation of biomolecules, and biological antioxidant potential (BAP), measuring the capacity of the plasma sample to reduce iron from ferric (Fe^3+^) to ferrous (Fe^2+^) form. The results of the d-ROMs test were expressed in arbitrary units called Carratelli units (U. Carr.), where 1 U. Carr. corresponds to 0.08 mg/100 mL H_2_O_2_; the results of the BAP test were expressed as μEq/L reduced iron. To evaluate humoral immune response, at the start of the trial (day 3) the cows were injected subcutaneously with 10 mg (5 mg per shoulder) keyhole limpet hemocyanin (KLH, Sigma-Aldrich, Milan, Italy) dissolved in 2 mL sterile saline solution and emulsified in an equal volume of incomplete Freund’s adjuvant. Another injection without adjuvant was repeated after two weeks. Antibody titre was evaluated, four times like the other measurements, by dosing the blood antibody on serum collected from the cows. The antibody titre in serum was determined following Braghieri et al. [9], and optical density (OD) at 450 nm was measured using an ELISA microplate reader (Bio-Rad, Model 680, Hercules, CA, USA). A skin test was performed, at the start (day 1) and the end (day 62) of the trial, to measure the cell-mediated immune response. Phytohemoagglutinin (PHA-P, Sigma-Aldrich; 1 mg dissolved in 1 mL sterile saline solution) was injected intradermally into the middles of 1.5-cm wide circles marked on shaved skin on the upper side of each shoulder. The skin fold thickness (mm) was measured with a calliper before and 24 h after the injection. For each cow, the increase in skin fold thickness (24-h thickness—preinjection thickness) was calculated using the mean of the two measurements taken from the shoulders.

The cows were milked mechanically twice a day, at 6:00 h and at 18:00 h. Individual milk yield was weighed, and samples were taken and analysed for lactose, fat, protein, casein, and somatic cell count (SCC) using infrared methods (Combifoss 6000; Foss Electric, Hillerød,, Denmark); urea by enzymatic methods using the difference in pH (CL-10 Plus; Eurochem, Rome, Italy); and total bacterial count (TBC; Bactoscan, Foss Electric).

To determine the FA composition of lyophilised individual milk samples (100 mg), the fat was extracted by direct methylation with 1 mL hexane and 2 mL 0.5 M NaOCH3 at 50 °C for 15 min, followed by 1 mL 5% HCl in methanol at 50 °C for 15 min, based on the bimethylation procedure [14]. Fatty acid methyl esters were recovered in hexane (1.5 mL). One microlitre of each sample was injected by autosampler into an HP 6890 gas chromatography system equipped with a flame-ionisation detector (Agilent Technologies, Santa Clara, CA, USA). Fatty acid methyl esters from all samples were separated using a 100 m (length), 0.25 mm (internal diameter), and 0.25 µm (film thickness) capillary column (CP-Sil 88; Chrompack, Middelburg, The Netherlands). The injector temperature was kept at 255 °C, and the detector temperature was kept at 250 °C, with an H2 flow of 40 mL/min, an air flow of 400 mL/min, and a constant helium flow of 45 mL/min. The initial oven temperature was held at 70 °C for 1 min, increased 5 °C/min to 100 °C, held for 2 min, increased 10 °C/min to 175 °C, held for 40 min, and finally increased 5 °C/min to a final temperature of 225 °C and held for 45 min. Helium was the carrier gas, with a head pressure of 23 psi and a flow rate of 0.7 mL/min (linear velocity = 14 cm/s). A solution of fatty acid methyl esters and hexane (Nu-Check-Prep, Elysian, MN, USA) was used to identify each FA. A standard mixture of methyl esters of CLA isomers (Sigma-Aldrich) and published isomeric profiles [15,16] were used to identify the rumenic acid (RA; CLA C18:2 c9 t11). Total FA was quantified using C23:0 (Sigma-Aldrich) as the internal standard, which was added at a concentration of 4 mg/g freeze-dried sample. Results were expressed as mg FA/100 g milk.

### 2.3. Statistical Analysis

Data were analysed statistically using the SAS 9.2 software. For individual data on behaviour, oxidative and immune status, milk production and FA composition, with the cow as the experimental unit, the fixed effects of feeding system (FS: Housing, grazing) and sampling day (SD, four levels) were assessed using a MIXED model for repeated measures, with SD as the repeated measure, and H cow as the repeated subject, regarded as a random error term. SCC and TBC values were transformed logarithmically (log10) before analysis.

Cell-mediated immune responses to the skin test were subjected to a General Linear Model (GLM) procedure evaluating the effects of FS, day of measurement (DM, day 1, day 62), and their interaction FS × DM.

Social interactions (expressed as n/cow) were subjected to GLM considering only the effect of FS.

## 3. Results

At the start of the trial, the available biomass at pasture was 2753 kg DM/ha, whereas at the end it was 1780 kg DM/ha. Both groups completely consumed the TMR and concentrates. The average hay intake of H cows was 4.8 kg/day per head.

Regarding behaviour, G cows spent longer eating (grazing + eating indoor) than H cows, which instead spent more time idling, walking, and drinking (Table 2). In addition, H cows spent more time in the outdoor area, where they stayed mostly lying down, rather than inside the barn. When G cows were outside, they spent more time standing; when they came back from pasture, they lay in the cubicles longer than H cows. G cows interacted more socially (Table 2), spending more time sniffing, licking, and rubbing one another affiliative manner, whereas H cows showed a greater amount of self-grooming.

Feeding system affected oxidative status (Table 3), as the G group exhibited a higher concentration of ROMs than the H group. In contrast, grazing did not increase antioxidant capacity, which was similar between the groups.

The antibody titre response that emerged from the measurement of serum OD (Table 3) did not differ between the groups, although the average OD of G cows was slightly higher than that of H cows. Indeed, daily testing revealed that the antibody titre of the G group was statistically higher than that of the H group only at first measurement (1.41 vs. 1.32 OD, *p* ≤ 0.05), which was nearest the time of the KLH antigen inoculation.

However, signs of a more efficient immune system in G cows emerged from the skin test with PHA-P (Table 3). The skin test performed at the start of the trial showed no differences between the groups, but when repeated at the end of the trial, the skin of G cows had not significantly changed, whereas that of H cows was thinner than both their initial result and that of G cows at the last test.

Grazing barley pasture did not affect milk yield, which was similar between the two groups (Table 4). However, it did improve milk quality in terms of an increase in fat and reductions in urea content, SCC, and TBC.

The fatty acid content in milk was influenced by the feeding system (Table 5). Saturated FA (C10, C12, C14, C16) were significantly higher in G milk, contrary to expectations. However, the milk of G cows had higher levels of FA beneficial to humans, such as RA (CLA, C18:2 c9 t11) and its precursor trans-vaccenic acid (VA, C18:1 t11), α-linolenic acid (ALA, C18:3 n3), and, as a tendency, all FA in the omega-3 series. The differences in milk FA content increased the saturated/unsaturated ratio in G cows but simultaneously reduced the omega-6/omega-3 ratio.

## 4. Discussion

### 4.1. Behaviour

Grazing cows spent twice as much time eating than cows fed exclusively indoors with unifeed, in line with other findings [10,17,18]. Commonly, animals at pasture carefully select grass, choosing the more tender and palatable plants or parts of plants; as a result, grazing animals tend to take smaller bites and eat slower for a longer time than animals fed in confinement [19]. Since H cows dedicated less time to eating, they had more available time, spent mostly idle, which can be a behavioural expression of boredom ([20]). As a consequence of green grass intake, G cows spent less time drinking water, consistent with Charlton et al. [18].

Contrary to a finding reported by Dohme-Meier et al. [21], G cows spent less time walking than the H group; however, in the current study grazing time has to be added to the amount of walking time, as cows at pasture, take slow and small steps forward as they ingest grass [10]. G cows did not rest lying down at pasture, presumably due to the short grazing time during which they almost continuously ate. However, once back indoors, they preferred to lie down in the cubicle, whereas H cows, which were kept permanently housed, chose to rest lying down in the outdoor area. Charlton and Rutter [10] reported that cows like to rest at night, and if weather permits and they can choose, they prefer to lay in the pasture, where there is a wider and more comfortable surface, rather than in the barn. However, in the present study, observations were made exclusively during the day, when the total time spent outdoors by the two groups was similar (66.8 vs. 64.0%, for the H and G groups, respectively).

Observations of sociopositive interactions (Table 2) showed that G cows spent more time sniffing and rubbing affiliatively or allogrooming; these behaviours reflect a greater degree of cohesion and reinforced social bonds among the cows, conditions that reduce aggression [22] and seem to be favoured by grazing. [23] suggested that allogrooming is an important behaviour with a fundamentally social function useful for the maintenance and stabilisation of relationships among cows. H cows showed more frequent self-grooming, an action that is often considered a stereotype, correlated with a state of boredom or increased stress [24]; nevertheless, the number of self-grooming activities was quite low, and may not have been due to stress in these cows, as also showed by their lower ROMs levels [25]. The frequency of aggressive interactions was not affected by the feeding system, and in both groups such interactions were quite infrequent.

### 4.2. Oxidative Status, and Immune Response

The oxidative status of cows, measured by ROMs and BAP levels, was affected by the feeding system. Contrary to expectations, grazing seemed to favour an increase in free radicals and did not increase the number of protective antioxidants. However, ROM levels in both groups were low, less than those reported in previous studies in which a negative energy balance, high milk production and early lactation phase affected oxidative status [26,27]. In the current study, neither group experienced any of these conditions, which suggests that ROM levels did not affect cow health or milk production, and thus implies negligible stress. Excluding the main factors that induce an increase in ROMs, such as beginning lactation, having a negative energy balance, thermal stress, high milk production, and disease, the higher ROM levels in the G group compared to the H group can be attributed to grazing, which can increase the production of free radicals [28].

The expected increase in the plasma level of antioxidant of G cows due to the high content of fat-soluble vitamins (vitamins A and E) in green forage [7] was not evident, presumably because the short duration of daily grazing or the well-known replacement effect with the TMR received indoors, limited the intake of antioxidant molecules from barley grass [29] at pasture.

The humoral immune response, following the injection of the KLH antigen, did not differ between the groups, although the OD of G cows tended to be higher than that of H cows on the first measurement, closest to inoculation. These results are partially comparable to those obtained by Braghieri et al. [5], who reported a better immune system in grazing young bulls in the middle of their study and similar values between the groups at the end of the trial. However, this longer duration of immune efficiency compared to the shorter one recorded in this study can be attributed to the higher time spent grazing (24 h) by young bulls [5]. Instead, the cell-mediated response to the skin test recorded in G cows expresses their better immune status, and is consistent with previous studies that have compared grazing and confined animals of different ruminant species [5,30,31,32].

### 4.3. Milk Production and FA Composition

The production responses recorded in this trial indicate that the availability of forage biomass at pasture and the short daily grazing time enabled dairy cows to produce comparable amounts of milk to cows kept permanently indoors and fed a diet based on concentrates and hay. These results are in line with Chapinal et al. [33] and Kennedy et al. [34], who in experiments with grazing cows obtained milk production similar to that of confined cows. Most similar experiments report greater quantities of milk produced by confined cows fed TMR, exclusively, especially when the cows are high yielding [35,36,37] or the feeding supplement does not appropriately support the nutritional needs of grazing cows.

From a qualitative point of view, grazing seems to have improved milk in terms of a higher fat content and a lower level of urea, SCC, and TBC. The fat content of the milk of G cows was higher, probably because of the increased forage/concentrate ratio in the diet comprising the green forage of the pasture. These cows ingested more fibre, which induced in the rumen the production of more acetic acid, a precursor of milk lipids synthesised in the udder. These results deviate from similar studies in which no difference in milk fat was found between cows fed TMR or at pasture [35,36] or in which the milk fat of the grazing cows was lower [34,38]. Over the entire experiment, the milk urea of grazing cows was lower than the confined group, but never lower than 28 mg/dL, which confirms that their intake of dietary crude protein did not limit milk production, in agreement with Fontaneli et al. [35]. The higher urea levels detected in H cows is probably related to the protein supplement given to these animals through the additional dose of soybean meal and faba bean [39]. The lower SCC and TBC in the milk of G cows than H cows can be attributed to grazing, in accordance with previous studies [35,40,41,42,43], in which grazing was found to be related to a lower incidence of subclinical mastitis and greater hygiene of the udder.

With regard to milk FA composition, contrary to most of the literature on the subject [44], in this study the content of saturated FA was higher in the milk of grazing cows, especially for palmitic acid (C16). This result, which is partly linked to the higher fat content in G milk, is in line with that of White et al. [45]. The greater amount of C16 found in the milk of G cows could be linked to the high concentration of palmitic acid in green barley [46] that was transferred to the milk. Indeed, palmitic acid is the third most represented FA in green grass, after the two polyunsaturated FA, ALA and linoleic acid [44]. Moreover, green forage in an advanced phenological stage, like that in this research, is characterised by a higher amount of palmitic acid, as reported by Coppa et al. [47].

The high level of saturated FA in the milk of G cows increased the saturated/unsaturated ratio; the few hours scheduled for grazing in this research probably did not allow G cows to ingest a sufficient amount of green grass to reduce this specific ratio in the milk. Nevertheless, the milk of grazing cows was characterised by a higher content of FA, which is beneficial for human health [48], such as RA (CLA, C18:2 c9 t11), its precursor VA (18:1 t11), and ALA (C18:3 n3). These results, which are in agreement with similar studies in which animals were fed green forage [5,49], show that grazing on barley grass contributed to an increase in the intake of linoleic acid and ALA. After being ingested, the latter are partly transferred directly to the milk and partly biodehydrogenated in the rumen to produce VA, which is subsequently desaturated by delta-9 desaturase in the mammary gland to produce RA, according to the well-known mechanism reported by Elgersma [44]. Finally, modification of FA content in the G milk led to a tendential increase in the level of total omega-3, and consequently a favourable reduction in the omega-6/omega-3 ratio to one more suitable for human health, as it was below the threshold (≤5) recommended by FAO/WHO [50].

## 5. Conclusions

Grazing cows had a more efficient immune system, which is crucial in preventing the onset of pathologies in livestock and therefore consistent with basic principles of an organic regime. However, contrary to expectations, a short grazing time did not increase antioxidants levels in dairy cows.

Moreover, the association between short grazing time and TMR offered indoors allowed the dairy cows raised under this organic regime to maintain milk production at the same level as cows permanently confined and fed TMR exclusively. The pasture even improved the quality of the milk by increasing its fat content and reducing the content of urea, SCC, and TBC.

The different feeding regimes of the cows influenced milk FA composition, with positive effects of grazing on the levels of some FA beneficial for human health, such as RA, the main CLA isomers, and ALA. Nevertheless, grazing on barley grass did not reduce the content of saturated FA in milk.

Therefore, according to the results obtained in this study, short-duration grazing can be used without negative consequences for organic cows. Even better, this option could reduce feed costs and improve the profitability of farming, producing foods with a lower environmental impact, which are increasingly requested by consumers.

## Figures and Tables

**Table 1:** Composition of diets as fed basis (kg/day per head) and chemical composition (% DM) of barley grass selected at pasture and feed offered to the experimental groups.

Item	Pasture	Total Mixed Ration (TMR) ^1^	TMR-Ingredients				
	Barley Grass		Hay	Soybean Meal	Faba Bean	Maize Grain	Barley Grain
Housing group ^2^		17.0	9.0	1.0	0.5	3.5	3.0
Grazing group	5 h/day	17.0					
DM, %	20.19	47.19	89.98	93.90	89.95	88.43	89.58
Crude protein	19.17	16.50	14.75	48.18	28.33	9.90	12.66
Ether extract	3.85	2.34	1.77	11.37	0.97	3.59	2.20
Ash	9.32	6.42	9.42	6.69	3.70	1.72	3.12
NDF	49.45	30.28	53.55	8.92	24.04	11.55	22.03
ADF	27.97	20.93	38.48	9.29	13.97	3.53	10.47
ADL	2.07	3.32	6.29	0.16	3.32	0.42	1.27

^1^ The TMR was supplemented with sodium-chloride, sodium-bicarbonate, and bacterial inoculum for unifeed stabilisation. ^2^ Cows in the housing group received a supplement of soybean (1.0 kg/day), faba bean (0.5 kg/day), and hay ad libitum, which was consumed at an average amount of 4.8 kg/day per head.

**Table 2:** Effect of feeding system on animal behaviour (% of observation time), social and aggressive interactions (n/cow).

Item	Posture	Housing	Grazing	SEM ^1^	*p*
Outdoors	Lying, %	34.2	10.5	1.93	<0.0001
	Standing, %	32.6	53.5	3.74	<0.0001
Indoors	Lying, %	5.52	15.9	2.06	0.0006
	Standing, %	27.6	20.1	2.29	0.1074
Grazing (GR), %		--	46.8		
Eating indoors (EI), %		31.9	16.0		
Eating (GR + EI), %		31.9	62.8	2.28	<0.0001
Drinking, %		1.92	0.46	0.145	0.0304
Ruminating, %		29.2	26.6	2.13	0.4996
Walking, %		5.58	2.15	0.492	0.0052
Idling, %		30.4	8.37	1.70	<0.0001
Affiliative interactions, n		1.43	3.18	0.344	0.0007
Aggressive interactions, n		0.25	0.56	0.121	0.1186
Self-grooming, n		2.98	1.75	0.322	0.0096

^1^ SEM = standard error of mean.

**Table 3:** Effect of feeding system on oxidative and immune parameters.

Item	Measure Unit	Housing	Grazing	SEM ^1^	*p*
Oxidative status					
ROMs	U. Carr.	61.7	87.3	8.10	0.0426
BAP	Log microEq/L	3.46	3.41	0.016	0.1082
Immune parameters					
Antibody titre	OD 450 nm	1.22	1.19	0.021	0.323
Skin fold thickness (mm) ^2^	day 1	4.17 a	4.10 ab	0.455	0.1269
	day 62	3.00 b	4.50 a		

^1^ SEM = standard error of mean. ^2^ Recorded at the start (day-1) and end (day-62) of the experiment after injection of PHA-P; the interaction between feeding system and day of measurement (FS × DM) was significant (*p* = 0.0315); among interaction means: a, b = *p* ≤ 0.05.

**Table 4:** Effect of feeding system on milk production.

Parameter	Measure Unit	Housing	Grazing	SEM ^1^	*p*
Milk	kg/day	25.6	25.5	1.47	0.9615
Fat	%	3.94	4.15	0.141	0.0419
Protein	%	3.59	3.49	0.127	0.5724
Casein	%	2.79	2.73	0.101	0.7059
Urea	mg/dL	36.1	29.5	0.706	0.0153
Lactose	%	4.96	4.97	0.069	0.9076
Somatic cells	log10 n/mL	5.13	4.89	0.127	0.0424
Total bacterial count	log10 n/mL	6.24	5.95	0.140	0.0335

^1^ SEM = standard error of mean.

**Table 5:** Effect of feeding system on the fatty acid (FA) content of individual cow milk (mg/100 g milk).

Fatty Acids	Housing	Grazing	SEM ^1^	*p*
C10	134.63	149.68	5.65	0.0645
C12	155.51	176.17	6.76	0.0346
C14	408.95	479.24	13.55	0.0005
C16	1043.06	1223.11	34.59	0.0005
C16:c1	38.11	45.48	2.14	0.0182
C18	257.15	256.65	10.55	0.9739
C18:1 *t11*, VA ^2^	11.18	12.27	0.36	0.0348
C18:1 *c9*, OA ^3^	526.56	554.09	20.44	0.3449
C18:2 n-6 *c9 c12*, LA ^4^	92.13	93.53	3.17	0.7572
C18:3 n-6 *c6 c9 c12*, GLA ^5^	4.46	5.08	0.23	0.0612
C18:3 n-3 *c9 c12 c15*, ALA ^6^	14.72	16.19	0.53	0.0441
CLA ^7^ C18:2 *c9 t11,* RA ^8^	5.96	7.04	0.28	0.0090
C20:5 n-3, EPA ^9^	2.94	3.16	0.14	0.2671
C22:5 n-3, DPA ^10^	1.34	3.05	0.81	0.1397
C22:6 n-3, DHA ^11^	3.22	3.34	0.13	0.5369
∑ saturated FA (SFA)	2377.55	2695.03	72.82	0.0031
∑ monounsaturated FA (MUFA)	695.55	735.96	24.85	0.2549
∑ polyunsaturated FA (PUFA)	163.14	166.30	4.75	0.6395
Σ unsaturated FA (UFA)	858.69	902.26	28.21	0.2792
SFA/UFA	2.79	3.05	0.0802	0.0217
∑ omega-6 FA ^12^	122.08	122.19	3.64	0.9838
∑ omega-3 FA ^13^	23.56	26.68	1.21	0.0730
omega-6/omega-3	5.26	4.78	0.15	0.0252

^1^ SEM = standard error of mean. ^2^ VA = trans-vaccenic acid; ^3^ OA = oleic acid; ^4^ LA = linoleic acid; ^5^ GLA = γ-linolenic acid; ^6^ ALA = α-linolenic acid; ^7^ CLA = conjugated linoleic acid; ^8^ RA = rumenic acid; ^9^ EPA = eicosapentaenoic acid; ^10^ DPA = docosapentaenoic acid; ^11^ DHA = decosahexaenoic acid; ^12^ ∑ omega-6 FA = C18:2 n-6 t9 t12; C18:2 n-6 *c9 t12*; C18:2 n-6 *t9 c12*; C18:2 n-6 *c9 c12* (LA); C18:3 n-6 *c6 c9 c12* (GLA); C20:2 n-6; C20:3 n-6; C20:4 n-6; C22:2 n-6; C22:4 n-6; ^13^ ∑ omega-3 FA = C18:3 n-3 *c9 c12 c15* (ALA); C20:3 n-3; C20:5 n-3 (EPA); C22:5 n-3 (DPA); C22:6 n-3 (DHA).
